# Changes in parafoveal and peripapillary perfusion after decompression surgery in chiasmal compression due to pituitary tumors

**DOI:** 10.1038/s41598-021-82151-1

**Published:** 2021-02-10

**Authors:** Ga-In Lee, Kyung-Ah Park, Sei Yeul Oh, Doo-Sik Kong

**Affiliations:** 1 Department of Ophthalmology, Samsung Medical Center, Sungkyunkwan University School of Medicine, 81 Irwon-ro, Gangnam-gu, Seoul, 06351 South Korea; 2 Department of Neurosurgery, Endoscopic Skull Base Surgery Clinic, Brain Tumor Center, Samsung Medical Center, Sungkyunkwan University School of Medicine, Seoul, South Korea

**Keywords:** Diseases, Neurology

## Abstract

We evaluated changes in parafoveal and peripapillary vessel density in chiasmal compression after decompression surgery using optical coherence tomography angiography (OCT-A). Sixty-two eyes with chiasmal compression for which preoperative and postoperative (4–6 months) OCT, OCT-A, visual field (VF), and comprehensive ophthalmic data were available, and 44 healthy eyes were evaluated. Vessel densities of the superficial retinal capillary plexus (SRCP), deep retinal capillary plexus (DRCP), and radial peripapillary capillary (RPC) segment were assessed using OCT-A. The postoperative measurements were compared with preoperative data. Preoperative peripapillary retinal nerve fiber layer, macular ganglion cell-inner plexiform layer thickness, and vessel densities of SRCP and RPC segments in patients’ eyes were significantly reduced compared to those of healthy controls (*P* < 0.0001, *P* < 0.0001, *P* = 0.0052, and *P* = 0.0085, respectively). Vessel densities were significantly decreased in the SRCP (*P* < 0.0001), DRCP (*P* = 0.0017), and RPC segments (*P* < 0.0001) after surgery compared to the preoperative values. Significant associations between the postoperative SRCP and DRCP vessel density changes and preoperative SRCP (*r* =  − 0.3195, *P* = 0.0114) and DRCP (*r* =  − 0.5165, *P* < 0.0001) vessel densities were found, respectively. There were also significant associations between postoperative SRCP vessel density changes and VF changes (*r* =  − 0.2586, *P* = 0.0424). These findings indicate that decreased perfusion around the optic nerve head and on the macula associated with chiasmal compression could further progress after decompression surgery. Further functional and longer-term clinical studies are needed to elucidate the clinical implications of these findings.

## Introduction

Visual dysfunctions including visual field (VF) defects such as temporal hemianopsia and decreased visual acuity due to pituitary tumors are common ophthalmic findings of chiasmal compression. These symptoms in pituitary tumors occur as a result of compression on the optic chiasm, and the chiasmal compression may lead to the retrograde degeneration of retinal ganglion cell bodies and axons. Retinal nerve fiber layer (RNFL)^1,2^ and ganglion cell complex (GCC)^3^ thinning on optical coherence tomography (OCT) in chiasmal compression have been reported in many previous studies. The thinning of these layers in eyes with hemianopsia due to chiasmal compression can persist after surgery^4–6^. One longitudinal study reported that the RNFL thickness and GCC area in patients with chiasmal compression were significantly reduced after three months postoperatively, while significant improvements in VF defects were observed after surgery^2^. Changes in retinal thickness measured by OCT have allowed us to estimate retrograde degeneration in vivo^1–3^. Recent advances in optical coherence tomography angiography (OCT-A) to reconstruct three-dimensional vascular structures in the retinal layers and peripapillary areas by auto-segmentation have enabled us to measure vessel density quantitatively and reproducibly^7^. OCT-A is widely used to identify vascular changes and understand the pathophysiology of various optic neuropathies such as glaucoma^8^, ischemic optic neuropathy^9^, and inflammatory optic neuropathy^10^. In primary open-angle glaucoma, a significant and progressive loss of vessel density compared to suspected glaucoma or healthy eyes has been reported^11^, suggesting that serial OCT-A measurements might allow us to sensitively detect glaucomatous changes^11^.

To date, there has been no study on the serial changes in OCT-A measurements in chiasmal compression before and after decompression surgery. Thus, the purpose of this study was to investigate changes in the preoperative and postoperative vessel density values in eyes with chiasmal compression using OCT-A.

## Patients and methods

This retrospective, longitudinal observational study involved 62 patients with chiasmal compression and 44 healthy controls at the Neuro-ophthalmology Department of Samsung Medical Center between April 2018 and May 2019. This study was conducted according to the tenets of the Declaration of Helsinki. The Institutional Review Board of Samsung Medical Center (Seoul, Republic of Korea) approved this study and waived the requirement for informed consent for patients with chiasmal compression. For the healthy controls, written informed consent was obtained prior to any ophthalmic examination.

Some of our patients participated in other studies in the same department^12,13^. However, previous data were not duplicated. All measurements were performed again, and new patients were included. The clinical diagnosis of chiasmal compression was made based on preoperative VF defects and/or decreased visual acuity and magnetic resonance imaging (MRI) evidence of tumor compression of the optic chiasm for all of the included patients. The onset time was defined as the time when the patient first noticed VF defects or experienced decreased vision related to chiasmal compression. In asymptomatic patients, the onset time was regarded as the time when VF defects were first detected upon formal ocular examination. All patients underwent trans-sphenoidal tumor resection and visited the clinic 4–6 months postoperatively. To assess the efficacy of chiasmal decompression, a postoperative MRI was performed, which is a routine postoperative procedure for all patients. The neurosurgeons and radiologists gather in a neurosurgery conference to evaluate the postoperative MRI results. The patients also underwent ophthalmic examinations including best-corrected visual acuity, visual field, OCT, and OCT-A preoperatively and 4–6 months postoperatively.

Forty-four disease-free subjects were recruited as controls from staff and healthy volunteers who had undergone routine eye examinations. None of these controls had a history of ocular or neurologic disease. The healthy controls were required to have normal visual acuity, normal intraocular pressure ≤ 21 mm, and normal optic discs. Patients or healthy controls with other ophthalmic diseases (glaucoma, a refractive error greater than 6.0 diopters of spherical equivalent (SE) or 3.0 diopters of astigmatism in either eye, amblyopia, epiretinal membrane, age-related macular degeneration, diabetic retinopathy, retinal artery/vein occlusion, or any optic neuropathy other than chiasmal compression), previous retinal surgery that could affect the thickness of the intra-retinal layer, and those who were diagnosed with known systematic/inflammatory diseases such as cancer and multiple sclerosis were excluded.

 The VF of each patient was tested with a Humphrey Field Analyzer using the 30-2 SITA-standard protocol (Humphrey 740 Visual Field Analyzer, Carl Zeiss Meditec Inc. Dublin, CA, U.S.A.). Only reliable VFs (≤ 33% false positives and false negatives; fixation losses < 20%) in two consecutive tests were analyzed. The mean deviation (MD) was used for the analysis. All subjects underwent fundus color photography and were scanned with a Cirrus HD-OCT (Carl Zeiss Meditec AG, Jena, Germany). The peripapillary RNFL (pRNFL) thickness was measured using an optic disc cube 200 × 200 protocol with Cirrus software. A recognition algorithm detected the inner and outer borders of the pRNFL. This protocol generated a cube of data via a 6-mm-square grid. A circle with a diameter of 3.46 mm was automatically centered on the optic disc. This analytical protocol yielded the average RNFL thickness, mapped four quadrants (superior, inferior, nasal, and temporal), and classified the results compared to an internal normative database. Only scans with a signal strength of ≥ 6 without motion artifacts were included. Using the macular Ganglion Cell Analysis algorithm, the thicknesses of the ganglion cell-inner plexiform layer were evaluated. The thicknesses of the GCIPL was automatically measured at various locations around the fovea (superior, temporal, nasal, and inferior).

**Figure 1:**
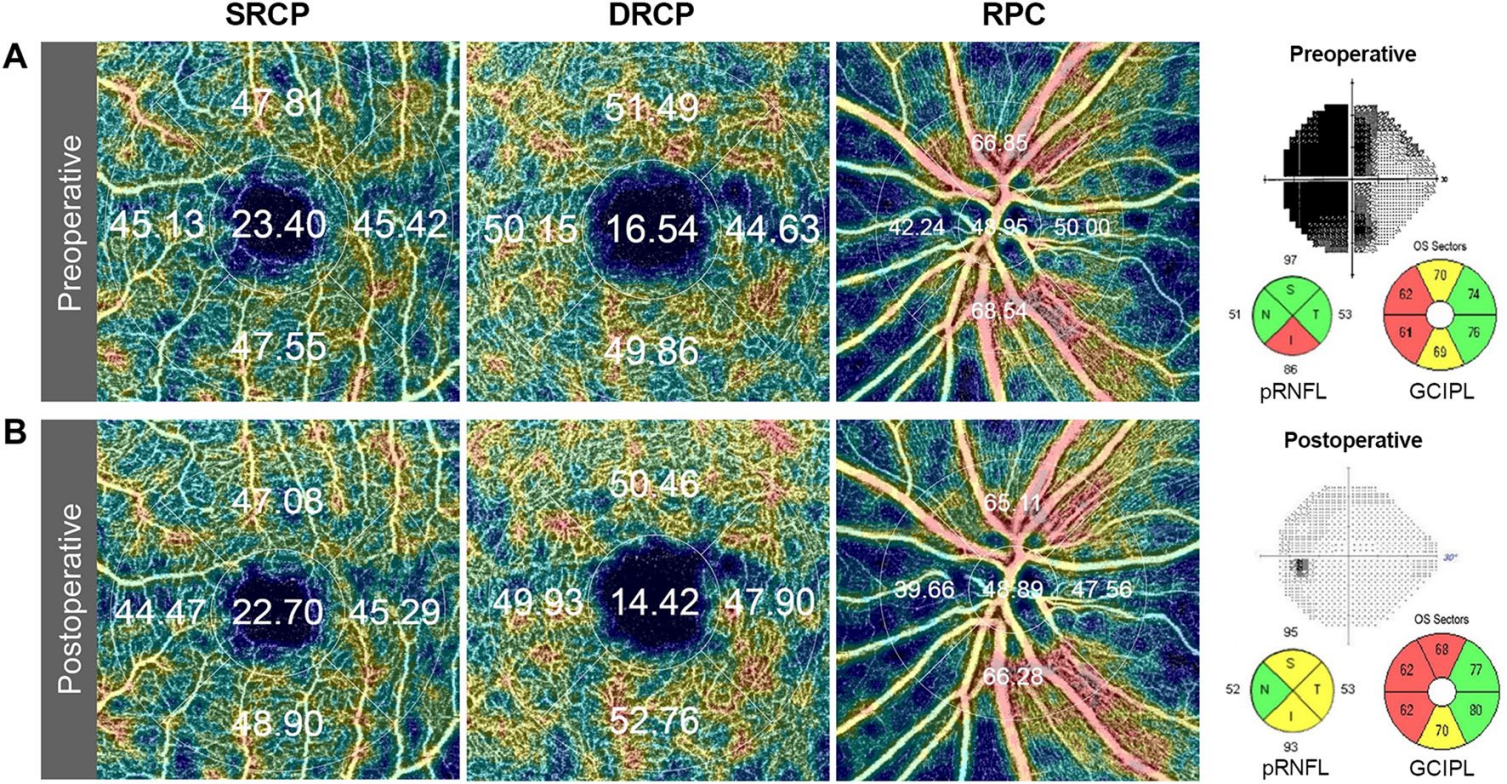
Representative (**A**) preoperative and (**B**) postoperative test results of a study patient with a pituitary adenoma. Optical coherence tomography angiography color-coded density map with image qualities of 72 in the superficial retinal capillary plexus (SRCP), deep retinal capillary plexus (DRCP), and radial peripapillary capillary (RPC) segments showing the automated measurement of vessel density with the percentage via auto-segmentation. The preoperative visual field of the patient showed temporal hemianopsia that completely recovered after surgery. The peripapillary retinal nerve fiber layer (pRNFL) and the ganglion cell-inner plexiform layer (GCIPL) thickness of the patient are presented pre- and postoperatively.

Retinal and peripapillary microvasculatures were analyzed using a Topcon OCT instrument (DRI OCT Triton Plus) for all patients and healthy controls. The Triton swept-source OCT uses a wavelength of 1050 nm with a scan speed of 100,000 A-scans per second. For each field scan, three repeated B-scans were obtained from 500 uniformly spaced locations. They were sequentially acquired to verify the repeatability of the vessel density measurement. Each B-scan consisted of 500 A-scans. The inter-scan time between the repeated B-scans was about 5 ms, accounting for the mirror scan duty cycle. The instrument uses an active eye-tracker that follows the fixation movement. It detects blinking and adjusts the scan position accordingly, thereby reducing motion artifacts during OCT-A image generation. Each patient underwent two imaging sessions consisting of a peripapillary scan (4.5 mm × 4.5 mm) centered on the optic disc and a 3.0 mm × 3.0 mm perifoveal scan centered on the macula. The superficial and deep retinal capillary plexuses were separated automatically via layer segmentation with the OCT instrument software (IMAGEnet 6 V.1.14.8538). The OCT performed automated segmentation and offered several preset reference boundaries for en- face projection, with a final rendered depth scale of 2.6 µm/voxel. The swept-source OCT-A evaluation included vessel density, foveal avascular zone area and the presence of vascular abnormalities such as dilated endings of the capillaries and the number of choriocapillaris flow voids. The superficial retinal capillary plexus (SRCP) extended from 3 µm below the internal limiting membrane (ILM) to 15 µm below the IPL, while the deep retinal capillary plexus (DRCP) extended from 15 to 70 µm below the IPL, according to the previously validated method of Park et al.^14^. The radial peripapillary capillary (RPC) segment extended from the ILM to the posterior boundary of the RNFL. Vessel density was defined as the percentage of the area occupied by vessels in a localised region. The software automatically fitted a 3 mm macular circle at the foveal center and generated vessel density for each layer with high repeatability and reproducibility^7,15^. Another 3 mm circle was manually fitted at the disc center. The pericentral vessel density was defined as the mean of four quadrants in the macular circle excluding the central circular area. Five areas (center, nasal, temporal, superior and inferior) dividing the center of the macula and disc are displayed (Fig. 1). The blood vessel density of each area was indicated as a percentage. All participants underwent both OCT-A and Cirrus HD-OCT imaging on the same day preoperatively or postoperatively. Eyes with a low image quality of < 40 and those showing a partial decrease in image intensity were excluded, Trained graders reviewed all images and excluded eyes with large eye movements during image capture reflected in motion artifacts involving more than three lines and any discontinuities in blood vessels in the OCT-A images. The location of the optic disc margin was reviewed for accuracy. The margin was adjusted manually as needed and confirmed by two graders (G.I.L and K.A.P).

Only a single eye (the one with the worse VF defect based on the MD of each patient with chiasmal compression and the right eye of the healthy controls) was selected for analysis. The data are presented as the mean ± standard deviation (SD). The best-corrected visual acuity (BCVA) was converted to a log MAR scale. The student’s t-test and Wilcoxon rank-sum test were used to compare age, SE, and the preoperative MD of the VF between the patients with chiasmal compression and healthy controls. Linear regression analysis was conducted after adjusting for age and SE to compare vessel densities, RNFL thickness, and GCIPL thickness between the patients and healthy controls. To compare the measurements before and after surgery, the generalized estimating equation was used after adjusting for age and SE. Spearman’s correlation coefficients were calculated to analyze the following ophthalmic factors: changes in intra-retinal layer thickness, VF defects before and after surgery, and preoperative vessel densities related to vessel density changes. A *P* value of less than 0.05 was considered statistically significant. All statistical analyses were performed with SAS version 9.4 (SAS Institute, Cary NC, U.S.A.).

## Results

### Comparison of patients with chiasmal compression and healthy controls

This study enrolled 62 eyes of 62 patients with chiasmal compression and 44 eyes of 44 healthy controls. These patients presented with chiasmal compression due to pituitary adenoma (n = 43, 69.4%), meningioma (n = 8, 12.9%), craniopharyngioma (n = 7, 11.3%), or Rathke’s cleft cyst (n = 4, 6.5%). The mean age of the patients was 51 ± 13 years (range 20 to 74 years) and the healthy controls were 49 ± 11 years (range 22 to 72 years). The mean duration of the visual symptoms was 5 ± 6 months (range 0.5 to 36 months). There were no significant differences in age, gender, or SE refractive errors between the two groups (Table 1). Both the preoperative pRNFL (average values and all quadrants except the nasal quadrant) and GCIPL (average values and all quadrants) were significantly thinner in the patient group than in the control group (all *P* < 0.001, pRNFL superior quadrant; *P* = 0.0066) except for the preoperative nasal quadrant pRNFL thickness (*P* = 0.0593), including the average values and four-quadrant values in linear regression analysis after adjusting for age and SE. Preoperative vessel densities of the SRCP (average values and all sectors except the temporal sector) and RPC segments (average values and temporal and nasal sector) were significantly decreased as compared to those of the healthy controls (Table 2).

**Table 1:** Baseline characteristics of patients with chiasmal compression preoperatively and healthy controls.

	Patients (*N* = 62)	Healthy controls (*N* = 44)	*P* value
Age (years)	51 ± 13	49 ± 11	0.5012*
Sex (M/F)	24/38	17/27	0.9939^†^
Spherical equivalent (diopters)	− 0.99 ± 1.73	− 1.37 ± 1.89	0.2389^‡^
Symptom duration (months)	5.71 ± 9.97	–	
Preoperative VF (MD)	− 8.03 ± 6.28	0.09 ± 1.16	** < 0.0001**^**‡**^
Preoperative BCVA (logMAR)	0.14 ± 0.23	0.0 ± 0.01	** < 0.0001**^**‡**^

Significant values are indicated in bold.

*N* numbers, *M* male, *F* female, *VF* visual field, *MD* mean deviation, *BCVA* best-corrected visual acuity.

*P value by Student’s t-test.

^†^*P* value by Chi-squared test.

^‡^*P* value by Wilcoxon rank sum test.

**Table 2:** Comparison of intraretinal layer thickness and parafoveal and peripapillary vessel densities in patients and healthy controls.

	Healthy controls (*N* = 44)	Patients (*N* = 62)		Comparison between preoperative values and healthy controls		Comparison between preoperative and postoperative values	
		Preoperative (*N* = 62)	Postoperative (*N* = 62)	Estimate (SE)	*P* value1	Estimate (SE)	*P* value2
VF (MD)		− 8.03 ± 6.28	− 3.34 ± 4.33			**4.70 (0.74)**	** < 0.0001**
BCVA (logMAR)		0.14 ± 0.23	0.04 ± 0.08			− **0.10 (0.03)**	**0.0003**
**pRNFL thickness (μm)**							
Average	100.24 ± 8.30	88.70 ± 11.60	85.40 ± 12.79	− **11.20 (2.02)**	** < 0.0001**	− **3.30 (0.77)**	** < 0.0001**
Temporal	77.45 ± 12.33	59.56 ± 14.54	59.66 ± 14.54	− **17.38 (2.62)**	** < 0.0001**	0.10 (1.20)	0.9358
Inferior	129.00 ± 14.28	115.24 ± 17.90	111.26 ± 19.28	− **13.34 (3.19)**	**0.0001**	− **3.98 (1.17)**	**0.0007**
Nasal	71.43 ± 14.09	66.90 ± 12.10	63.18 ± 12.63	− 4.91 (2.57)	0.0593	− **3.73 (1.38)**	**0.0068**
Superior	122.43 ± 13.31	113.10 ± 18.62	107.50 ± 21.32	− **9.17 (3.30)**	**0.0066**	− **5.60 (1.47)**	**0.0001**
**GCIPL thickness (μm)**							
Average	84.29 ± 5.95	73.87 ± 7.93	73.82 ± 7.73	− **10.61 (1.41)**	** < 0.0001**	− 0.03 (0.39)	0.9467
Temporal	84.31 ± 6.14	78.27 ± 5.60	77.94 ± 7.06	− **6.26 (1.17)**	** < 0.0001**	− 0.31 (0.40)	0.4378
Inferior	81.67 ± 6.29	72.65 ± 8.03	72.03 ± 8.66	− **9.20 (1.44)**	** < 0.0001**	− 0.58 (0.43)	0.1749
Nasal	84.32 ± 5.71	70.65 ± 11.93	69.41 ± 12.47	− **13.72 (1.91)**	** < 0.0001**	− 1.19 (0.64)	0.0627
Superior	84.33 ± 6.58	73.00 ± 9.41	71.82 ± 10.53	− **11.37 (1.65)**	** < 0.0001**	− **1.16 (0.58)**	**0.0434**
**SRCP (%)**							
Average	48.62 ± 2.35	47.34 ± 2.06	45.95 ± 2.04	− **1.22 (0.43)**	**0.0052**	− **1.40 (0.20)**	** < 0.0001**
Temporal	47.38 ± 2.91	47.23 ± 2.43	46.19 ± 2.57	− 0.15 (0.53)	0.7809	− **1.04 (0.33)**	**0.0014**
Inferior	49.99 ± 3.45	47.93 ± 3.21	46.10 ± 3.25	− **2.01 (0.65)**	**0.0027**	− **1.84 (0.37)**	** < 0.0001**
Nasal	47.62 ± 2.40	46.00 ± 2.80	44.73 ± 2.81	− **1.57 (0.51)**	**0.0028**	− **1.27 (0.30)**	** < 0.0001**
Superior	49.48 ± 2.94	48.21 ± 2.77	46.77 ± 3.01	− **1.14 (0.54)**	**0.0362**	− **1.44 (0.34)**	** < 0.0001**
**DRCP (%)**							
Average	49.43 ± 2.20	48.75 ± 1.98	47.79 ± 2.24	− 0.57 (0.40)	0.1572	− **0.97 (0.31)**	**0.0017**
Temporal	47.33 ± 2.90	47.31 ± 2.95	46.84 ± 2.72	0.07 (0.58)	0.9001	− 0.47 (0.39)	0.2332
Inferior	51.49 ± 4.55	50.04 ± 3.51	48.55 ± 4.06	− 1.34 (0.77)	0.0867	− **1.49 (0.59)**	**0.0116**
Nasal	48.32 ± 2.65	47.58 ± 3.12	46.80 ± 2.96	− 0.64 (0.58)	0.2726	− **0.79 (0.37)**	**0.0339**
Superior	50.55 ± 3.12	50.07 ± 3.33	48.95 ± 3.32	− 0.35 (0.64)	0.5897	− **1.12 (0.45)**	**0.0121**
**RPC (%)**							
Average	58.32 ± 2.54	56.15 ± 4.77	**54.58** ± 5.28	− **2.00 (0.75)**	**0.0085**	− **1.57 (0.28)**	** < 0.0001**
Temporal	48.5 ± 5.39	43.98 ± 7.12	43.17 ± 7.31	− **4.29 (1.15)**	**0.0003**	− 0.81 (0.56)	0.1493
Inferior	67.89 ± 5.63	67.67 ± 6.28	65.93 ± 7.69	0.03 (1.18)	0.9815	− **1.74 (0.54)**	**0.0013**
Nasal	50.72 ± 4.55	48.55 ± 6.26	46.61 ± 7.29	− **2.32 (1.10)**	**0.0379**	− **1.95 (0.57)**	**0.0006**
Superior	66.15 ± 4.33	64.39 ± 7.64	62.60 ± 7.32	− 1.42 (1.25)	0.2586	− **1.79 (0.67)**	**0.0075**

Significant values are indicated in bold.

*N* numbers, *VF* visual field, *MD* mean deviation, *BCVA* best corrected visual acuity, *pRNFL* peripapillary retinal nerve fiber layer, *GCIPL* ganglion cell-inner plexiform layer, *SRCP* superficial retinal capillary plexus, *DRCP* deep retinal capillary plexus, *RPC* radial peripapillary capillary.

*P values by linear regression with adjustment for age and spherical equivalent.

^†^*P* values by generalized estimating equation analysis with adjustment for age and spherical equivalent.

*P* value 1: linear regression analysis between preoperative patients and healthy controls.

*P* value 2: linear regression analysis between preoperative and postoperative patients.

### Comparison of OCT and OCT-A parameters before and after surgery

The vessel densities, pRNFL thickness, and macular GCIPL thickness in the patients with chiasmal compression after decompression surgery are summarized in Table 2. When the pre- and post-operative ophthalmic parameters were compared, VF defects (estimate (standard error), 4.70 (0.74), *P* < 0.0001) and visual acuity (− 0.10 (0.03), *P* = 0.0003) significantly recovered after decompression surgery. pRNFL thickness (average, − 3.30 (0.77), *P* < 0.0001; inferior, − 3.98 (1.17), *P* = 0.0007; nasal, − 3.73 (1.38), *P* = 0.0068; superior, − 5.60 (1.47), *P* = 0.0001) and superior quadrant GCIPL thickness (− 1.16 (0.58), *P* = 0.0434) significantly decreased after surgery. The vessel densities of the SRCP (average, − 1.40 (0.20), *P* < 0.0001; temporal, − 1.04 (0.33), *P* = 0.0014; inferior, − 1.84 (0.37), *P* < 0.0001; nasal, − 1.27 (0.30), *P* < 0.0001; superior, − 1.44 (0.34), *P* < 0.0001), DRCP (average, − 0.97 (0.31), *P* = 0.0017; inferior, − 1.49 (0.59), *P* = 0.0116; nasal, − 0.79 (0.37), *P* = 0.0339; superior, − 1.12 (0.45), *P* = 0.0121), and RPC segments (average, − 1.57 (0.28), *P* < 0.0001; inferior, − 1.74 (0.54), *P* = 0.0013; nasal, − 1.95 (0.57), *P* = 0.0006; superior, − 1.79 (0.67), *P* = 0.0075) were further reduced significantly after decompression surgery in patients with chiasmal compression.

### Associations between changes in vessel density and other ocular parameters

**Figure 2:**
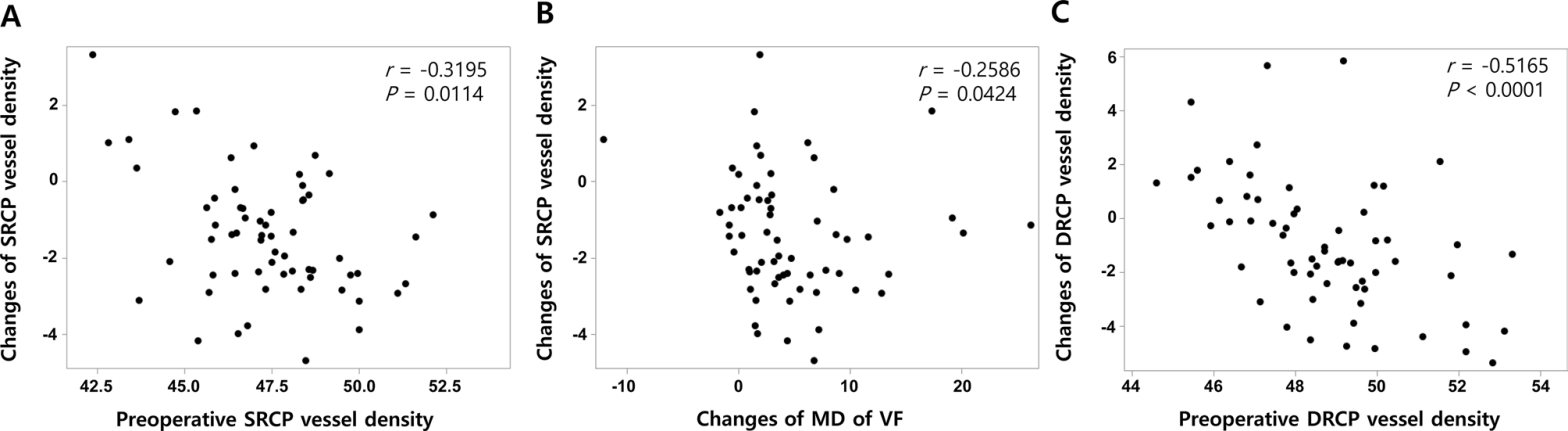
Scatterplots showing significant associations between vessel density changes and preoperative vessel denstiy and VF changes in patients with chiasmal compression. (**A**) Negative correlation between vessel density changes in the superficial retinal capillary plexus (SRCP) pre- and postoperatively and preoperative SRCP vessel density in patients with chiasmal compression (*r* =  − 0.3195, *P* = 0.0114); (**B**) negative correlation between vessel density changes in the SRCP and changes in the mean deviation of the visual field pre- and postoperatively (*r* =  − 0.2586, *P* = 0.0424); (**C**) negative correlation between vessel density changes in the deep retinal capillary plexus (DRCP) pre- and postoperatively and preoperative DRCP vessel density (*r* =  − 0.5165, *P* < 0.0001).

A greater postoperative decrease in SRCP vessel density was associated with lower preoperative baseline vessel density in the SRCP (*r* =  − 0.3195, *P* = 0.0114) in Spearman’s correlation analysis. More decrease in vessel density in the DRCP was also associated with lower preoperative baseline vessel density in the DRCP (*r* =  − 0.5165, *P* < 0.0001) in Spearman’s correlation analysis (Table 3, Fig. 2).

**Table 3:** Association analysis between vessel density changes and preoperative vessel density and structural and functional changes.

	Correlation coefficient	*P* value*
**Average SRCP vessel density changes**		
MD of VF changes	− **0.2586**	**0.0424**
pRNFL thickness changes	0.1279	0.3218
GCIPL thickness changes	− 0.1760	0.1786
Preoperative SRCP vessel density	− **0.3195**	**0.0114**
**Average DRCP vessel density changes**		
MD of VF changes	− 0.0975	0.4510
pRNFL thickness changes	0.1202	0.3519
GCIPL thickness changes	− 0.2209	0.0899
Preoperative DRCP vessel density	− **0.5165**	** < 0.0001**
**Average RPC vessel density changes**		
MD of VF changes	− 0.0437	0.7360
pRNFL thickness changes	0.1836	0.1532
GCIPL thickness changes	0.0778	0.5548
Preoperative RPC vessel density	0.0344	0.7908

Significant values are indicated in bold.

*MD* mean deviation, *VF* visual field, *pRNFL* peripapillary retinal nerve fiber layer, *GCIPL* ganglion cell-inner plexiform layer, *SRCP* superficial retinal capillary plexus, *RPC* radial peripapillary capillary.

*P values by Spearman’s correlation.

The association between OCTA measurements and visual functions were also analyzed. The greater the postoperative decrease in the SRCP vessel density, the worse the postoperative VF outcomes (*r* =  − 0.2586, *P* = 0.0424). We also found that lower postoperative vessel density in the SRCP was associated with worse postoperative visual acuity (*r* =  − 0.2980, *P* = 0.0187), and lower postoperative vessel density in the RPC segment was associated with worse postoperative VF (*r* = 0.4804, *P* = 0.0001) and visual acuity (*r* =  − 0.2705, *P* = 0.0335) (Table 4).

**Table 4:** Association analysis between postoperative vessel density and functional outcomes.

Postoperative parameters	Correlation coefficient	*P* value*
**Average SRCP vessel density**		
MD of VF	0.2257	0.0778
BCVA	− **0.2980**	**0.0187**
**Average DRCP vessel density**		
MD of VF	0.1801	0.1613
BCVA	− 0.0584	0.6520
**Average RPC vessel density**		
MD of VF	**0.4804**	**0.0001**
BCVA	− **0.2705**	**0.0335**

Significant values are indicated in bold.

*MD* mean deviation, *VF* visual field, *BCVA* best corrected visual acuity, *SRCP* superficial retinal capillary plexus, *RPC* radial peripapillary capillary.

*P values by Spearman’s correlation.

## Discussion

It is well known that axonal injury causes changes in the inner and outer retinal layer thicknesses in patients with chiasmal compression^5,6^. These changes are due to retrograde degeneration that leads to ganglion cell loss in the medial retina, which was proven by animal experiments^16,17^. In this study, pRNFL thickness showed a significant decrease after decompression surgery for a chiasmal tumor despite improved visual acuity and VF. This result is also consistent with results of previous human studies^1,5^.

Damage to the ganglion cell axon of the optic nerve could lead to retrograde degeneration, and it may reduce the metabolic demand on the anterior optic nerve, leading to changes in peripapillary and retinal vascular structures. Previously, we reported a postoperative reduction in intraretinal vessel density in 36 patients with chiasmal compression compared to 35 healthy controls in a cross-sectional study^12^. That study reported the association between visual function and postoperative intraretinal vessel density^12^. In a subsequent study with a larger sample size of 57 patients, we reported the relationship between preoperative intraretinal vessel density and postoperative functional visual outcomes^13^. However, no study has reported postoperative intraretinal vascular changes compared to preoperative values in chiasmal compression. The present study attempted to investigate changes in parafoveal and peripapillary vessel densities before and after surgery using an OCT-A device known to be capable of quantitative measurement of intraocular vessel density. The patients in the present study already showed significant decreases in parafoveal and peripapillary vessel densities preoperatively. Although visual acuity and VF recovered significantly after the surgery, postoperative vessel density was found to be significantly decreased. Damage to ganglion cells due to delayed retrograde degeneration might further decrease metabolic activity and affect nutrient demand and supply, leading to secondary changes in retinal and peripapillary vascular structures. In the present study, there was a significant association between changes in vessel density and preoperative baseline vessel density in the SRCP and DRCP. The lower density of blood vessels in the SRCP and DRCP, the greater the decrease in postoperative vascular density.

Regarding the association between OCT-A measurements and visual function, a significant association between changes in the superficial macular vessel density and VF changes was found. Greater superficial macular vessel density was associated with worse postoperative VF recovery. Also, we found that lower postoperative superficial macular vessel density and peripapillary vessel density were associated with worse postoperative visual outcomes. These results were consistent with a previous study showing a significant association between peripapillary and macular vessel density and the corresponding VF loss^18^.

The present study had several potential limitations. First, our data were obtained 4 to 6 months after decompression surgery. Thus, the follow-up period was relatively short. Further studies are needed to evaluate longer-term changes in intra-retinal vessel density in chiasmal compression after decompression surgery. Second, vessel density was measured using OCT-A in which the angiographic signal was based on movement. However, many other factors such as blood flow velocity, morphology, and alterations in the vascular endothelial barrier can compromise the measurement of perfusion. Therefore, we cannot exclude false-positive findings due to technical and methodological issues. Last, this study was confined to a single center with a population of Asian ethnicity. Some results may not be valid in other ethnic groups.

In conclusion, we investigated preoperative and postoperative vessel density alterations measured with OCT-A for eyes with chiasmal compression along with changes in the OCT parameters. Postoperative intraretinal vessel density changes were found to be associated with preoperative intraretinal vessel density. Changes in intraretinal vessel density were also associated with VF changes. These findings suggest that parafoveal and peripapillary perfusion in chiasmal compression is affected both preoperatively and postoperatively and that intraretinal vascular changes are associated with functional outcomes.

## Data Availability

All datasets generated during and/or analyzed during the current study are available from the corresponding author upon reasonable request.
